# Correction to “Real‐World Practitioner Perceptions of CARTITUDE‐4 Results for Patients With Previously Treated Multiple Myeloma”

**DOI:** 10.1002/jha2.70050

**Published:** 2025-05-19

Balanean A, Baird S, Dulka B, Jennings‐Zhang L, Bone RN, Jeune‐Smith Y, et al. Real‐world practitioner perceptions of CARTITUDE‐4 results for patients with previously treated multiple myeloma. eJHaem. 2024;5:1154‐64. https://doi.org/10.1002/jha2.1047

In Figure 3 the “After” percentages for two response options were inadvertently transposed in the figure, creating an inconsistency with the description provided in the text.

Specifically:

The “After” result for the response option “I favor CAR T‐cell therapy in earlier lines (e.g., 2L or 3L settings)” should be 55% (not 31%).

The “After” result for the response option “I want real‐world and long‐term outcomes data on CAR T‐cell therapy in any line” should be 31% (not 55%).

We apologize for this error.

